# Simultaneous synthesis and integration of nanoscale silicon by three-photon laser direct writing†

**DOI:** 10.1039/d2na00845a

**Published:** 2023-01-20

**Authors:** Kai Li, Zhijun Luo, Heng Jiao, Zongsong Gan

**Affiliations:** a Wuhan National Laboratory for Optoelectronics, Huazhong University of Science and Technology Wuhan Hubei 430074 China ganzongsong@hust.edu.cn; b Key Laboratory of Information Storage System Ministry of Education of China, Huazhong University of Science and Technology Wuhan Hubei 430074 China; c Shenzhen Huazhong University of Science and Technology Research Institute Shenzhen Guangdong 518057 China

## Abstract

Typical fabrication processes of compact silicon quantum dot (Si QD) devices or components entail several synthesis, processing and stabilization steps, leading to manufacture and cost inefficiency. Here we report a single step strategy through which nanoscale architectures based on Si QDs can be simultaneously synthesized and integrated in designated positions by using a femtosecond laser (532 nm wavelength and 200 fs pulse duration) direct writing technique. The extreme environments of a femtosecond laser focal spot can result in millisecond synthesis and integration of Si architectures stacked by Si QDs with a unique crystal structure (central hexagonal). This approach involves a three-photon absorption process that can obtain nanoscale Si architecture units with a narrow line width of 450 nm. These Si architectures exhibited bright luminescence peaked at 712 nm. Our strategy can fabricate Si micro/nano-architectures to tightly attach to a designated position in one step, which demonstrates great potential for fabricating active layers of integrated circuit components or other compact devices based on Si QDs.

## Abstract 2

Nanoscale silicon architectures can be simultaneously synthesized and integrated by a laser method. Due to extreme environments of the focusing laser spot, silicon quantum dots were instantaneously synthesized to assemble nano-architectures.

## Graphical abstract

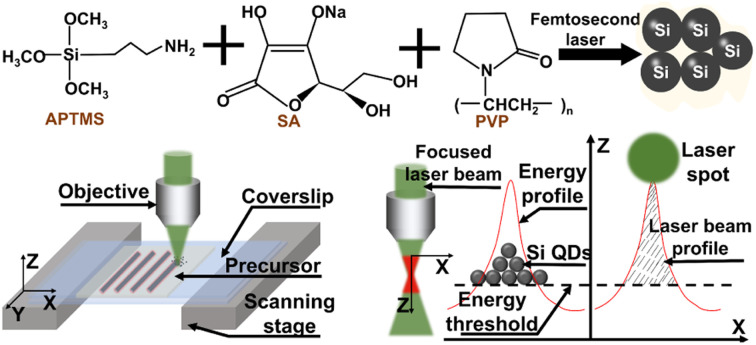

## 1. Introduction

Silicon quantum dots (Si QDs) have great potential for compact devices, due to their elevated temperature stability,^1,2^ distinctive optical and electrical characteristics,^1–3^ rich resource abundance,^4,5^ non-toxic properties^4^ and nanoscale footprint.^1,2^ Different applications of Si QDs have been widely reported with a variety of fabrication methods. For example, Si QD transistor arrays have been fabricated with a standard integration manufacturing process to perform as nanoscale necessary hardware for silicon-based chips, owing to the unique single electron spin properties of Si QDs.^2^ Si QDs fabricated with techniques of chemical vapor deposition, magnetron sputtering and electrochemical dissolution have high-brightness light emission (photoluminescence quantum yields ∼ 24%) properties.^6^ This makes Si QDs have significant advantages in micro-light emitting diodes (micro-LEDs).^7^ Furthermore, the Si QDs prepared by a standard Schlenk technique can be applied in devices of viable biomedical imaging, due to their long fluorescence lifetime.^8^ However, all of these Si QD devices were typically fabricated by a step-by-step process with synthesis and integration separated, which requires a long processing time, represents a complex process flow and leads to unstable device properties.^9^ Moreover, it is also difficult to arbitrarily integrate these Si QDs at the micro/nano-scale for device functionality. Therefore, the key challenge for the fabrication and integration of Si QD devices is developing a cost-effective and processes simplified strategy.

In this work, we propose a scalable strategy for simultaneously synthesizing and integrating nanoscale Si in an ambient environment and demonstrate the potential of femtosecond laser direct writing to fabricate active layers of semiconductor components. In contrast to typical step-by-step fabrication, femtosecond laser direct writing can construct desirable nanoscale Si architectures attached to different substrates (silicate glass cover slides, calcium fluoride substrates, and copper mesh carbon films) in a single step. To perform the strategy, high-quality Si QDs were synthesized on a millisecond time scale while a femtosecond laser was focused on the interface between a substrate and a transparent precursor. Subsequently, these Si QDs were stacked to form nanoscale three-dimensional (3D) Si architectures on the designated areas of a substrate. The formation of Si architectures has been confirmed with its elemental composition and crystal structure. We can repeatedly fabricate Si basic building blocks to assemble into arrays or print them even larger, to create scalable components. Our work shows that femtosecond laser direct writing can significantly reduce complexity and improve flexibility compared with other traditional manufacturing techniques of micro/nano-devices.

## 2. Experimental section

### 2.1. Reagents and materials

Sodium ascorbate (SA, 99%), (3-aminopropyl)trimethoxysilane (APTMS, 97%), polyvinylpyrrolidone (PVP, MW ∼ 40 000), and ascorbic acid (AA, 99%) were purchased from Aladdin Reagent Co. Ltd. All chemicals were used without further purification in this work.

### 2.2. Precursor solution

The precursor solution of Si QDs was prepared *via* a method reported in the literature with minor modifications.^10^ Briefly, 0.06 g SA and 0.01 g AA were mixed with 10 mL distilled water, and the mixture was kept in an argon gas atmosphere for 10 minutes to remove air. Then, 1.2 mL mixture and 1 mL APTMS were injected into 4 mL distilled water, and continuously stirred for 15 minutes at room temperature. After that, 1 g PVP was added under vigorous stirring. After PVP was completely dissolved, the final product was stored away from light.

### 2.3. Si architecture fabrication

**Fig. 1:**
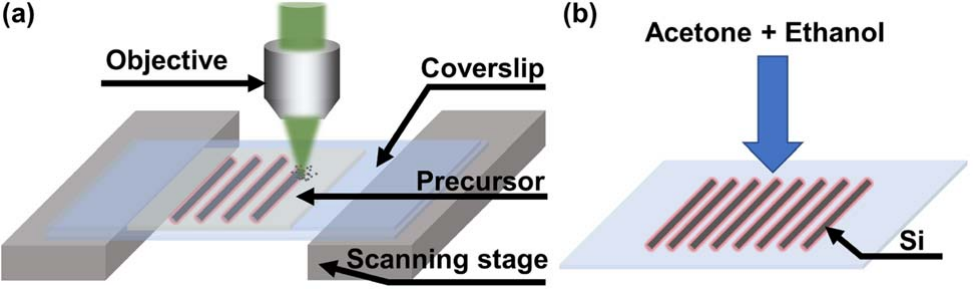
Illustration of simultaneous synthesis and integration of silicon with femtosecond laser direct writing. (a) Laser direct writing Si micro/nano-architectures on a substrate. (b) Developing process with acetone and ethanol.

Three-dimensional Si architectures at the micro/nano-scale were fabricated and integrated in one step by a multi-photon femtosecond laser direct writing method. Briefly, a drop (∼50 μL) of precursor solution was sandwiched between two coverslips. A femtosecond laser with a 532 nm wavelength (see the ESI† for details) was focused on the interface between the bottom coverslip and the precursor through an objective lens with a high numerical aperture (100×, NA = 1.4, Olympus). 3D Si micro/nano-architectures were simultaneously synthesized and integrated by moving the positions of the femtosecond laser focal spot with a scanning stage (563.3CD, Physik Instrumente), as illustrated in Fig. 1a. The final product was immersed in a developing liquid (acetone and ethanol, 1 : 1) for 5 minutes to clean the precursor (Fig. 1b).

## 3. Results and discussion

**Fig. 2:**
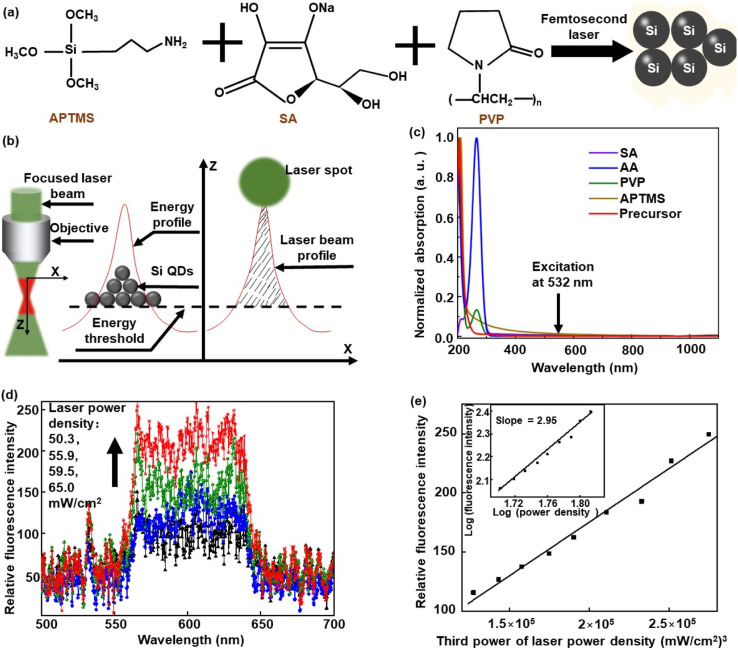
The mechanisms for simultaneous synthesis and integration of the micro/nano-Si architectures. (a) Schematic representation of the Si QD synthesis, the femtosecond laser decomposed APTMS to produce Si QDs and coagulation under the action of PVP. (b) Schematic representation of the formation of Si architectures stacked by Si QDs, and energy of the laser focused spot crossed the photochemical reaction threshold in the precursor, leading to the generation of Si QDs and coagulation with PVP. (c) Absorption spectra of SA, AA, PVP, APTMS, and the precursor solution of Si. (d) The fluorescence of Si precursor solution excited with a 532 nm laser. (e) The fluorescence intensity *vs.* the third power of laser power density, and the inset shows a double logarithm plot of the fluorescence intensity *vs.* laser power density.

A new strategy of simultaneous synthesis and integration requires that the chemical composition of precursors can produce Si nanocrystals to assemble arbitrary architectures under femtosecond laser irradiation. To enable high precision, multi-photon absorption should be the dominant process with respect to single-photon absorption, because the multi-photon absorption of the precursor restricts the excitation to the focal region of the laser beam only and avoids spillover or proximity effects common and unavoidable in single-photon absorption. Thus, it is necessary for the precursor to have high transmittance at the femtosecond laser wavelength. Based on these considerations, APTMS, SA, AA, and PVP were dissolved in distilled water to prepare a precursor of transparent gel. The APTMS and SA acted as a silicon source and a reducing agent, respectively. The gel solution of PVP can simulate a microgravity environment to provide auxiliary conditions for rapid crystallization (Fig. S1, ESI†). The AA was only used to regulate the pH of the precursor and hardly participates in the chemical reaction. As illustrated in Fig. 2a, the major compositions of the precursor can produce Si QDs under femtosecond laser illumination. The premise of Si QD synthesis is that the femtosecond focusing spot energy needs to cross the threshold of the photochemical reaction of the precursor. Subsequently, these Si QDs were coagulated with PVP gel to form 3D architectures whose shape is dependent on the femtosecond laser energy profile with a Gaussian distribution (Fig. 2b). The center of a femtosecond laser spot has the highest energy, and the energy of the edge is lowest. While the photon energy of the femtosecond laser crosses the reaction threshold of the precursor, the Si QDs of the laser spot center were first synthesized and integrated, and then grown towards the edge. Additionally, the precursor and all its compositions are transparent in liquid solutions (Fig. S2†). Therefore, the light from visible to near-infrared wavelengths cannot be absorbed *via* a single-photon process in the precursor (Fig. 2c). From absorption spectra, it is obvious that multi-photon absorption of 532 nm light is required to absorb the incoming femtosecond laser. Under the irradiation of a 532 nm laser focus spot, the transparent precursor can emit fluorescence which may be excited by the way of multi-photon absorption (Fig. 2d). And we can see that the fluorescence intensity is proportional to the third power of the laser power density (Fig. 2e). The inset in Fig. 2e presents the double logarithm plots of fluorescence intensity *vs.* laser power density, and the slope is 2.95. This indicates that the Si precursor upon 532 nm femtosecond laser excitation is certainly a multi-photon process, and the order is three. The multi-photon absorption process is caused by ultra-high energy density at the focal spot of the femtosecond laser.^11^ Only the multi-photon absorption can realize the energy transferred from the 532 nm laser light to the precursor, because the precursor is transparent for the 532 nm femtosecond laser and can hence not absorb energy *via* a single-photon process. Under 532 nm femtosecond laser irradiation, the photon energy was transferred from the femtosecond laser to the precursor by the way of multi-photon absorption. The reaction activity of the precursor is greatly improved compared with that of the precursor without photon energy. Subsequently, Si QDs were rapidly synthesized to assemble into a desirable architecture.

**Fig. 3:**
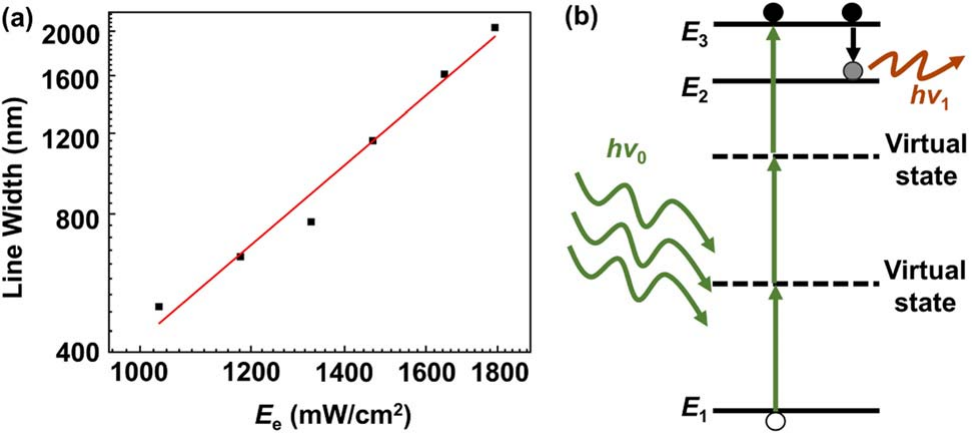
The order of the multi-photon process. (a) The line width of Si architectures *vs.* optical power density. (b) Schematic of three-photon absorption in precursor solution.

With a fixed scanning speed (*V* = 2 μm s^−1^) to fabricate Si line-architectures, the relationship between the laser power density (*E*_e_) and the line width (*W*_L_) of Si architectures was investigated (Fig. 3a). According to these data, we can confirm that the multi-photon process is a three-photon process rather than a two-photon process. The order of the multi-photon process can be calculated by using the equation:^12^ log *W*_L_ ∝ (*N*/*α*) × log *E*_e_ (see the ESI† for more details), where *α* (*α* ≥ 1) and *N* are the coefficient constant and the order of the multi-photon process, respectively. According to the data acquired from Fig. 3a, the order of the multi-photon process is *N* ≥ 2.6, which suggested that an effective process of three-photon absorption occurred in experiment (Fig. 3b). Due to three-photon absorption in the precursor, high-precision micro/nano-Si architectures can be integrated. Under femtosecond laser illumination with an intensity of 1030 mW cm^−2^, the fabricated Si architectures have a minimum line width of 450 nm. Under femtosecond laser illumination with an intensity of 1466 mW cm^−2^, the fabricated Si architectures have high quality and good uniformity (Fig. S3 and S4†). The line width size of architectures almost remains unchangeable and the fluctuation is within 10%. In order to ensure high quality Si micro/nano-device fabrication, the femtosecond laser illumination intensity was fixed at a value of 1466 mW cm^−2^ and the Si line-architecture fabrication scanning speed was fixed at a value of 2 μm s^−1^, if not specified otherwise. According to the moving speed of the scanning stage, the crystallization time of the Si line-architectures is estimated on a millisecond time scale (see the ESI† for details).

**Fig. 4:**
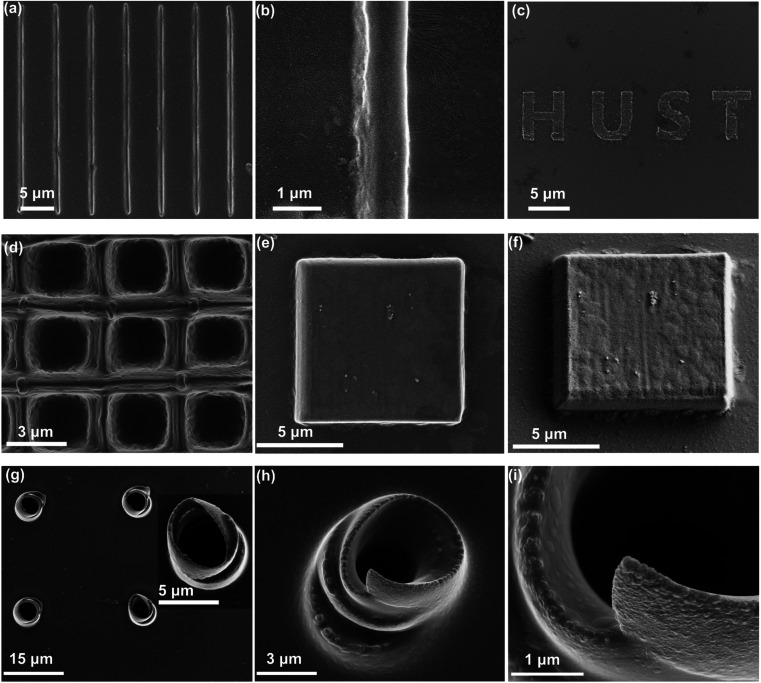
The SEM images of Si architectures. (a) The SEM image of Si line-architectures. (b) The SEM image of a single Si line. (c) The SEM image of 2D Si architectures of “HUST”. (d) The SEM image of the frame Si architecture. (e and f) The top view and oblique view SEM images of the cube Si architecture, respectively. (g) The top view SEM image of upright helix Si architectures, and inset shows an enlarged upright helix. (h and i) The oblique view SEM images of an upright helix.

The arbitrary micro/nano-Si architectures were simultaneously synthesized and attached to substrates, and their morphology was characterized by using a field emission scanning electron microscope (SEM), as shown in Fig. 4. The SEM characterization results show that all the Si line-architectures are uniformly integrated on a substrate and have high compactibility (Fig. 4a and b). Some agglomerates on the substrate can also be observed. They could be attributed to the PVP residues after acetone and ethanol development (Fig. S5†). Through accurate movement of the scanning stage, we can control the separation or assembly of line units to achieve nanoscale 2D or 3D Si architecture integration in any designated areas. To demonstrate the complex fabrication ability in a designated area, 2D Si architectures of “HUST” words were exhibited, as shown in Fig. 4c. Meanwhile, 3D Si architectures of frames and cubes were fabricated, as shown in Fig. 4d and e, respectively. Through the oblique view of the Si cube, the regular 3D morphology can be observed more clearly (Fig. 4f). We also built helical architectures to show the true three-dimensionality (Fig. 4g–i and S6†). All of the arbitrary Si architectures fabricated by this way are tightly attached to their substrate (Fig. S7†). These results indicate that Si micro/nano-architectures fabricated by our strategy are particularly suitable for active layer formation of compact components.

**Fig. 5:**
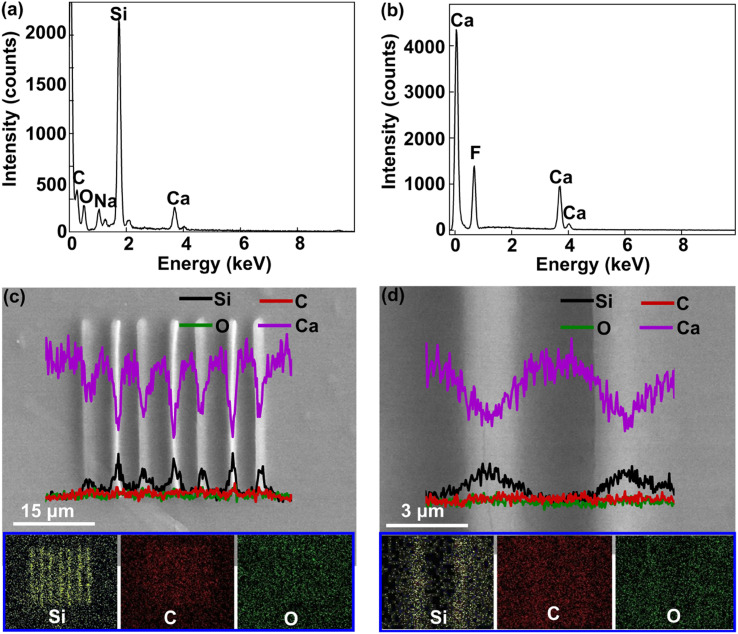
Characterization of Si architecture formation. (a) EDS of the fabricated Si architectures. (b) EDS of the calcium fluoride substrate. (c and d) The SEM images of Si lines, and their corresponding EDS line-scan curves with different chemical elements, and the insets at bottom show EDS mapping of the fabricated Si architectures.

It is crucial to investigate the formation of Si architectures fabricated by three-photon absorption femtosecond laser direct writing. The energy dispersive spectrum (EDS) of the fabricated Si architectures was investigated (Fig. 5a). The results show that Si element accounts for the highest proportion inside the Si architectures. It illustrates that the integrated architectures are mainly composed of Si elements. To make a comparison, pure calcium fluoride slides were specially used as substrates instead of coverslips with silicate glass. Fig. 5b shows that the substrates with calcium fluoride do not contain any Si element. It indicates that the Si elements detected by EDS originated from the fabricated architectures rather than the substrate. The elements of oxygen (O) and carbon (C) can also be identified in the Si architectures. They can be ascribed to the organic residual after acetone and ethanol development. The EDS line-scan profiles of elemental distributions indicate that the line-architectures are rich in Si element, and the element content reaches the highest proportion in the center position of the fabricated line (Fig. 5c and d). The element distribution profile of calcium (Ca) is opposite to that of Si, owing to the fact the Ca is originated from the bottom coverslip substrate. The element contents of C and O have little change from the fabricated line region to the non-fabricated blank region. As shown in the inset at the bottom of Fig. 5c and d, the EDS mapping results also demonstrate that Si element is concentrated on the fabricated lines, while C and O elements are evenly distributed in the whole observation area. These reveal that the fabricated architectures are formed from Si element rather than carbide or oxide of silicon.

**Fig. 6:**
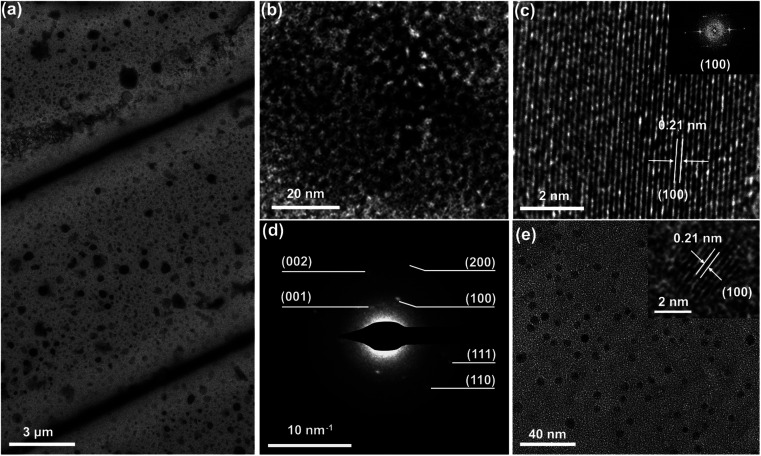
Crystallization structures of the fabricated Si architectures. (a) TEM images of Si line-architectures. (b) TEM image of a local enlarged Si architecture. (c) HR-TEM image of Si architectures, and the inset shows the FFT pattern of its corresponding area. (d) SAED image of Si architectures. (e) TEM images of Si QDs in the edge of Si architectures, and the inset shows the HR-TEM image of an individual Si QD.

The morphology, crystallinity and crystal structure of the fabricated Si architectures were characterized by using a high-resolution transmission electron microscope (HR-TEM), as shown in Fig. 6. The fabricated Si architectures are formed by compact accumulation of high-density Si QDs (Fig. 6a and b). HR-TEM and fast Fourier transform (FFT) images suggest that the fabricated Si architectures have good crystallinity (Fig. 6c). Good crystallinity and compact accumulation reveal the excellent reliability and photoelectric characteristics of the fabricated Si architectures. The measured lattice spacing inside the fabricated Si architectures is 0.21 nm, which is identical to the (100) lattice plane of central hexagonal structured Si (Fig. 6c). The crystal structure of the fabricated Si architectures was further confirmed by the selected area electron diffraction (SAED) pattern (Fig. 6d). The main SAED diffraction rings of the fabricated Si architectures can be readily indexed to central hexagonal structured Si (see the ESI† for details). These results confirmed that the fabricated Si architectures were constructed by dense accumulation of Si QDs with a central hexagonal crystal structure. We could then observe the morphology of the Si QDs in the area between Si architectures. As shown in Fig. 6e and S8,† the Si QDs have uniform size with an average diameter of 3.8 nm, and the inset shows the HR-TEM image of an individual Si QD with a diameter of 3.5 nm. The lattice spacing of individual Si QDs is consistent with that of the fabricated Si architectures. The measured lattice spacing (∼0.21 nm) is different from that (∼0.3 nm) of Si QDs synthesized by a traditional solution method.^4,13,14^ Compared with the literature, we have confirmed that the synthesized Si QDs have a central hexagonal crystal structure instead of the reported central cubic structure.^4,13,14^ Even if the precursor is almost consistent, the crystalline system of Si architectures prepared by our method is inconsistent with the reported results of traditional solution synthesis.^13^ This indicates that the nonlinear process induced by a high-energy femtosecond laser can lead to the generation of a unique crystalline system that is different from conventional solution synthesis processes. It might inspire us to think about the reasons for the different crystalline systems resulting from the femtosecond laser preparation and traditional method.

**Fig. 7:**
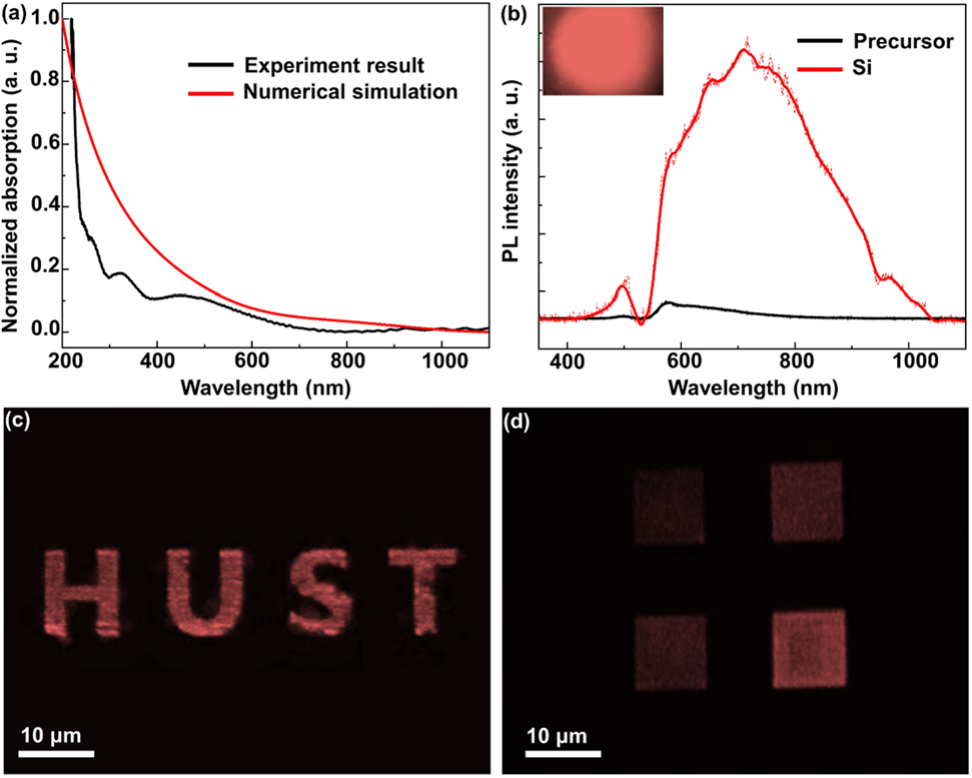
Optical properties of the fabricated Si architectures. (a) The absorption spectra of Si architectures. (b) The PL emission spectra of the Si architectures and their precursor, and the inset shows an image of Si architecture luminescence monitored by using a charge coupled device in PL testing. (c and d) The fluorescence microscopy images of Si architectures.

As illustrated in Fig. 7a, the fabricated Si architectures have strong broad-band absorption at the violet-visible light wavelength band, which is consistent with other reported results.^15^ Indeed, the experimental result of the absorption spectrum is in good agreement with the FDTD numerical simulation of Si QDs (see the ESI† for more simulation details). The photoluminescence (PL) spectra of the fabricated Si architectures were obtained under an excitation of a 532 nm wavelength (Fig. 7b). The fabricated Si architectures exhibit a broadband emission with the peak wavelength at 712 nm. This result is in line with other reported results.^4^ The emission wavelength peak at 580 nm can be attributed to the luminescence of the precursor. Emission wavelength peaks at 651, 755 and 965 nm are caused by O or C surface terminated Si QDs.^16^ These terminations originate from APTMS. The absence of a peak at 532 nm is ascribed to the filter, which reduces the measurement influence of the excitation laser source. Absorption and PL spectra can further confirm that the fabricated Si architectures were constructed from femtosecond laser synthesized and integrated Si QDs. Additionally, the inset of Fig. 7b shows that the fabricated Si architectures exhibit bright luminescence monitored by using a charge coupled device under 532 nm wavelength laser excitation. The fluorescence microscopy images also demonstrated that Si architectures have bright luminescence under 488 nm wavelength excitation (Fig. 7c and d). Fig. 7d shows that four 3D Si blocks with successively higher heights have different luminescence intensities. The higher block architectures have more Si QDs, which lead to more intense luminescence. After thermal treatment, these Si architectures do not lose luminous properties, which demonstrated that the Si QDs have good temperature stability (Fig. S9†). The luminescent Si architecture can be repeatedly fabricated and assembled into arrays by the strategy of three-photon femtosecond laser direct writing. The proposed technique of simultaneous synthesis and integration of nanoscale Si has potential applications in the field of micro-LEDs.

## 4. Conclusions

To summarize, nanoscale Si architectures were simultaneously synthesized and integrated in designated areas by a scalable three-photon femtosecond laser direct writing method. As the three-photon absorption of the precursor restricts the excitation to the focal region of the laser beam only, the desirable Si architectures were obtained with a narrow line width of 450 nm. The durable Si architectures were integrated with densely stacked high quality Si QDs, which were synthesized by femtosecond laser irradiation within milliseconds. As a proof of concept, the elemental analyses and crystal structures of the fabricated Si architectures were demonstrated, and the crystallization time was estimated. We also demonstrated that these Si architectures can be repeatedly fabricated and assembled into brightly luminous arrays by femtosecond laser direct writing. The strategy is significantly flexible and convenient compared with other step-by-step methods. The process is not limited to Si QDs and Si architectures, and other functional semiconductor materials that can be synthesized by composition modulations are also expected suitable candidates. This work provides an alternative for processing techniques of integrated circuit components or small size devices like micro-LEDs.

## Author contributions

This work was carried out with contributions from all authors. Kai Li searched the databases, screened the literature, designed the experiment, performed the research, extracted the data, assessed the included studies' quality and wrote the first draft of the manuscript. The author Zhijun Luo managed the statistical analysis of the analyses and revision. The author Heng Jiao provided experimental support and data validation. The corresponding author Zongsong Gan conceptualized the study, appraised the manuscript, and supervised the project. All authors read and approved the final manuscript.

## Conflicts of interest

The authors declare no conflicts of interest.

## Supplementary Material

NA-005-D2NA00845A-s001
