# Supplementary material for: Simultaneous synthesis and integration of nanoscale silicon by three-photon laser direct writing
Source: Nanoscale Adv. 2023 Jan 20;5(5):1299–306. doi: 10.1039/d2na00845a (PMC9972563; doi:10.1039/d2na00845a)
Supplement: NA-005-D2NA00845A-s001 [file NA-005-D2NA00845A-s001.pdf]

Electronic Supplementary Material (ESI) for Nanoscale Advances.
This journal is © The Royal Society of Chemistry 2023

Supporting Information

Simultaneous Synthesis and Integration of Nanoscale Silicon by Three-photon Laser Direct Writing

*Kai Li, Zhijun Luo, Heng Jiao, Zongsong Gan**

Wuhan National Laboratory for Optoelectronics, Huazhong University of Science and Technology, Wuhan, Hubei 430074, China

Key Laboratory of Information Storage System Ministry of Education of China, Huazhong University of Science and Technology, Wuhan, Hubei 430074, China

Shenzhen Huazhong University of Science and Technology Research Institute, Shenzhen, Guangdong 518057, China

*** Corresponding Author**

Zongsong Gan, E-mail: ganzongsong@hust.edu.cn

1. Parameters of femtosecond laser

The Si architectures were fabricated by a 532 nm femtosecond laser (200 fs pulse duration, 50 MHz repetition rate, FemtoPower FP532-fs, Fianium). The average power range of Si architectures fabrication is ≥ 150 mW.

2. Characterization

The morphology and chemistry component of the samples were characterized by using a field emission scanning electron microscope (Hitachi S4800). The crystal structure of the samples was characterized by using a high-resolution transmission electron microscope (FEI Talos F200X). Optical absorption spectra of the samples were recorded by using a UV-Vis-NIR spectrophotometer. Photoluminescence spectra were recorded by using a high-resolution spectrometer (LASERTECH, Aurora 4000) with a 532 nm laser as excitation source installed on a home-made imaging system. The luminescence image in Photoluminescence testing was monitored by a charge coupled device (WAT-902H2, Watec). The fluorescence images were taken by a fluorescence microscopy (K1-Fluo, Nanoscope System) with an excitation wavelength of 488 nm. All the optical measurements were performed in ambient environment (25°C, atmospheric environment, relative humidity 60%).

3. The order of multi-photon process

The order of multi-photon process can be calculated by the following equation:[1]

$$\log R \propto \frac{N}{\alpha} \log P \tag{1}$$

where R, P, α ($\alpha \geq 1$) and N are polymerization growth rate, laser power, coefficient constant and the order of multi-photon process, respectively. The $R = W_L \times t$ and $P = E_e \times S$, where W_L, t, E_e and S are linewidth, exposure time, laser power density and laser spot area. The t and S are fixed values in our experiment. Therefore, the equation (1) can be deformed:

$$\log W\mathrm{L} \propto \frac{N}{\alpha} \log E\mathrm{e} \qquad (2)$$

4. Estimation of crystallization time

As the laser spot moves one spot distance from a position, the Si nanocrystals at the position had been synthesized successfully. Therefore, we assumed crystallization time is the time that the spot passes through a diameter distance of spot. The crystallization time is calculated using the following equation:

$$t_c = \frac{d}{V} \qquad (1)$$

Where the t_c is the crystallization time, d is the diameter of the laser spot, V is the scanning stage ($V = 2$ μm/s). The d can be calculated by Abbe resolution:[2]

$$d = \frac{\lambda}{2\mathrm{NA}} \qquad (2)$$

Where the λ is the wavelength of femtosecond laser (532 nm), NA is the numerical aperture of objective (NA = 1.4). The diameter of the laser spot is about 190 nm. According to estimation, the t_c is about 95 millisecond. In fact, the real crystallization time should be shorter.

5. Parameter information of crystal structure

The fabricated Si architectures can be indexed to central hexagonal structure Si, space group: P6/mmm [191], a = b = 2.463, c = 2.326, $\alpha = \beta = 90^{o}$, $\gamma = 120^{o}$, PDF-#: No.47 – 1186.

6. FDTD numerical simulation

The FDTD numerical simulation of absorption could be by the equation: A = 1 – T – R, where A is absorption, T is transmission and R is reflection.

In the FDTD simulation experiment, we first set up a spherical silicon quantum dot (QD) model, the material of QD used in the simulation was Si (Silicon)-Palik. The simulation region: x = -12 nm to 12 nm， y = -12 nm to 12 nm, z = -8.43224 nm to 8.04626 nm. The boundary conditions: $x_{min/max}$ bc are periodic, $y_{min/max}$ bc are periodic, $z_{min/max}$ bc are perfectly matched layer (PML). The light source type selects plane wave, the wavelength is set to 200 nm -1100 nm. The monitors type selects frequency-domain field and power. Then, two monitors are placed in front and behind the QD model to measure the transmission and reflection, respectively.

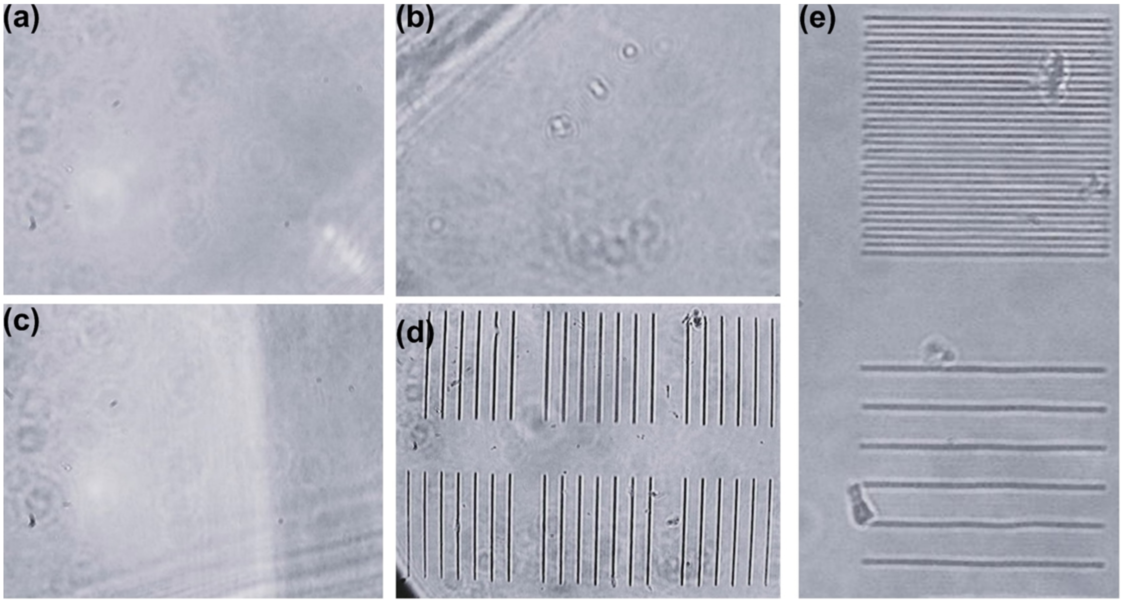

Figure S1. Optical pictures of different samples taken during the fabrication process from CCD camera. (a) PVP solution, (b) precursor without PVP, (c) precursor with 0.1g PVP and (d) precursor with 1g PVP, (e) precursor without AA. Only the gel precursor with excess PVP (1g) can be crystallized to assemble Si architectures successfully. The precursor with a few PVP or without PVP, are all not to form effective gel contribute to rapid crystallization. The precursor without AA still can be crystallized to assemble Si architectures. The AA was only used to regulate pH of the precursor and hardly participates in the chemical reaction.

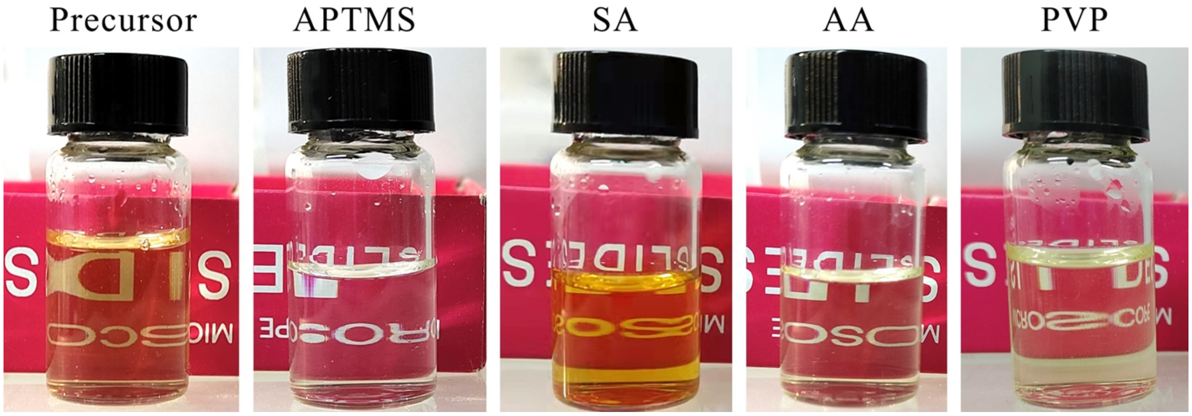

Figure S2. The photographs of precursor, APTMS, SA, AA and PVP aqueous solutions. all solutions demonstrate high transparency for background word.

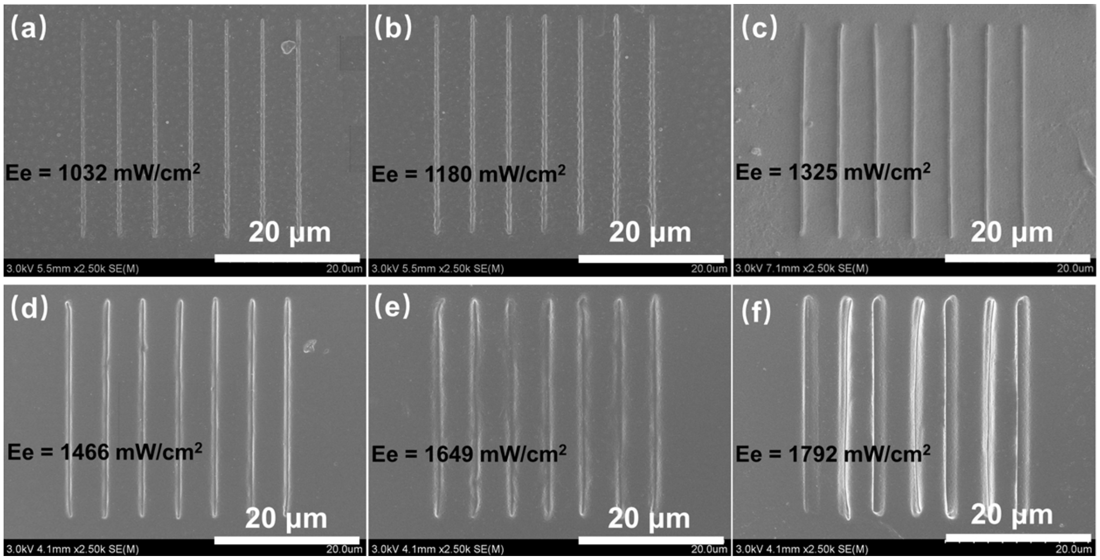

Figure S3. The SEM images of Si line-architectures under different optical power density.

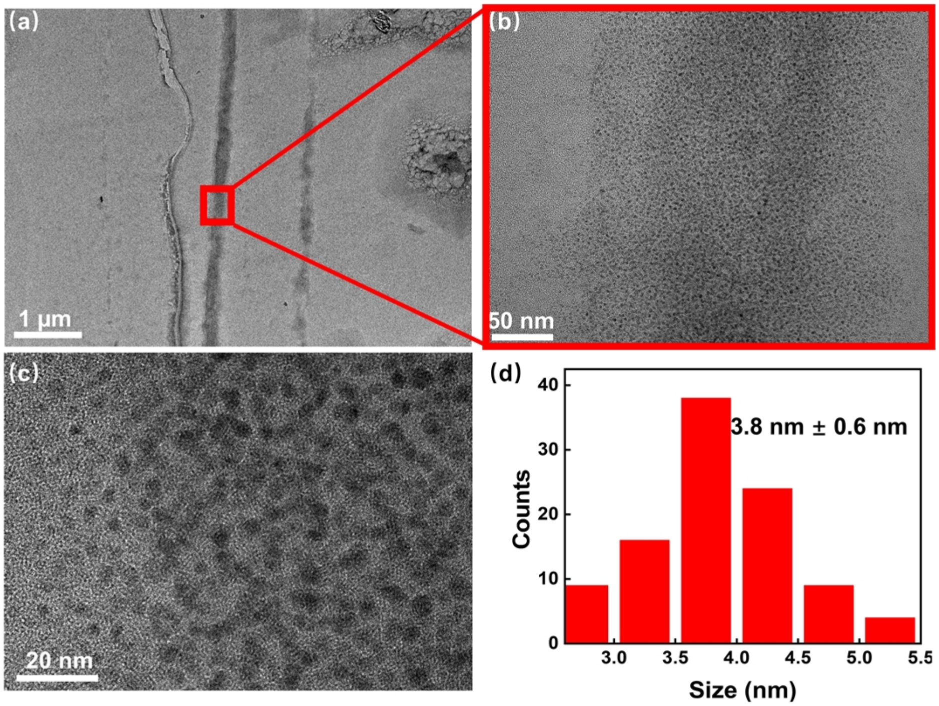

Figure S4. (a - c) The TEM images of Si architectures stacked by Si QDs. (d) The size distribution of Si QDs in architectures. These Si QDs exhibit high crystal quality, and have uniform size with an average diameter of 3.8 nm ± 0.6 nm.

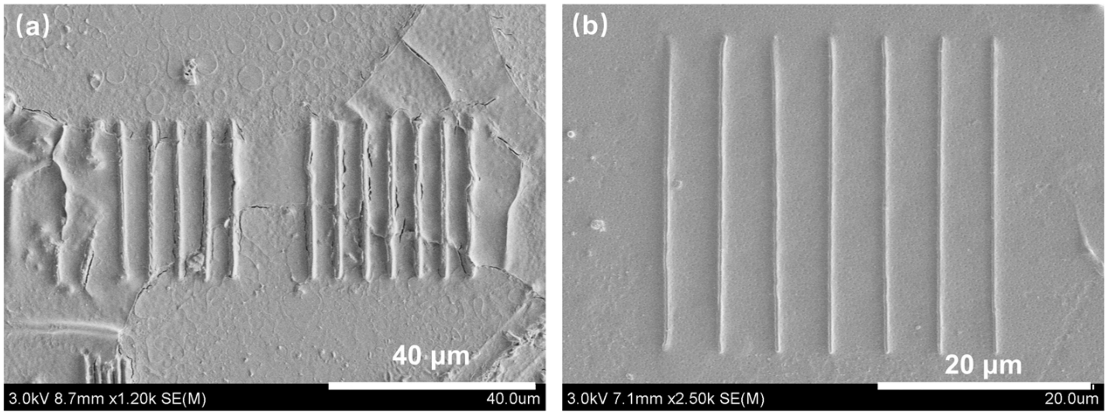

Figure S5. The SEM images of Si line-architectures (a) before and (b) after development. PVP gel would remain on the lines surface before development. After development, there might still be a small amount of PVP gel.

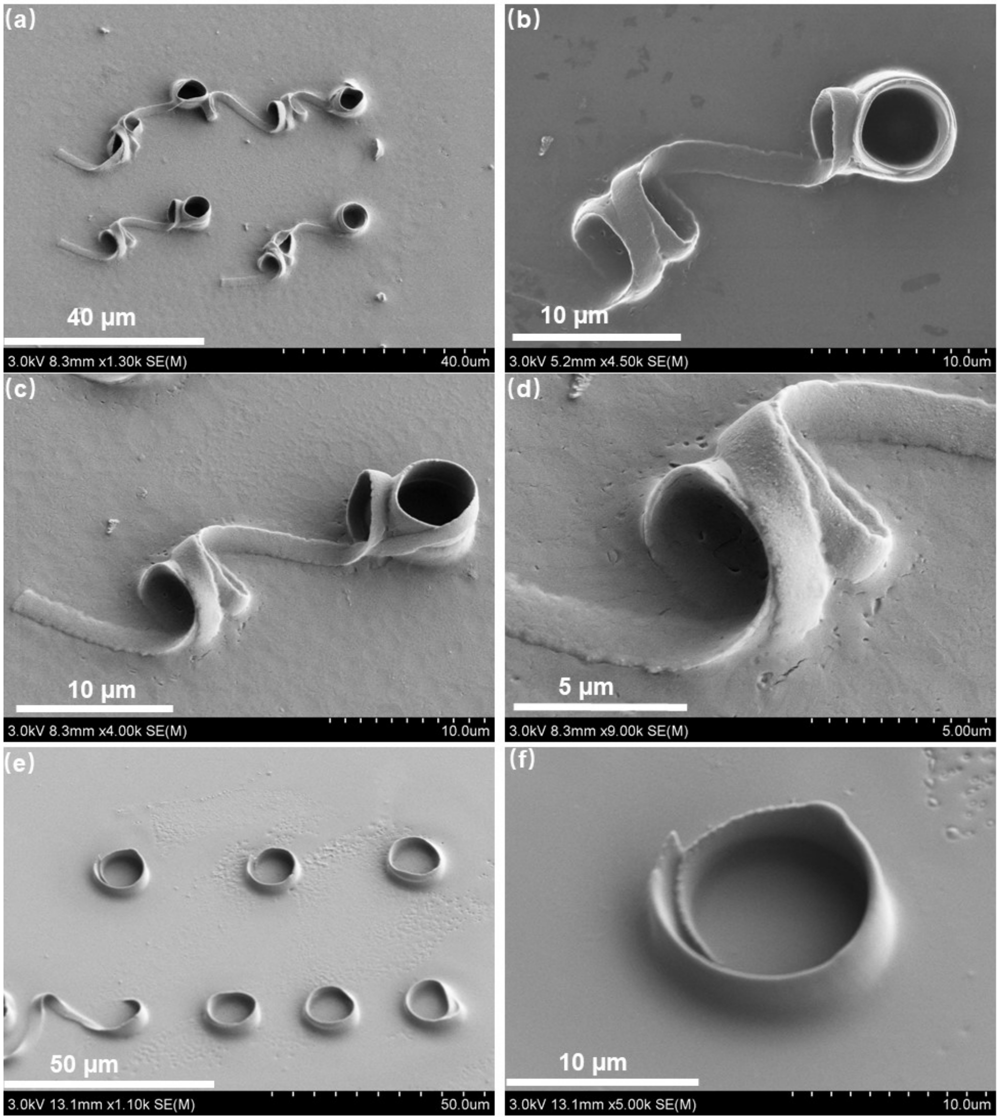

Figure S6. (a) The top view SEM images of Si helices. (b) The top view SEM images of a Si helix. (c) The oblique view SEM images of a Si helix. (d) Enlarged SEM image of the local area. (e) The oblique view SEM images of Si upright helices. (f) The oblique view SEM image of a Si upright helix.

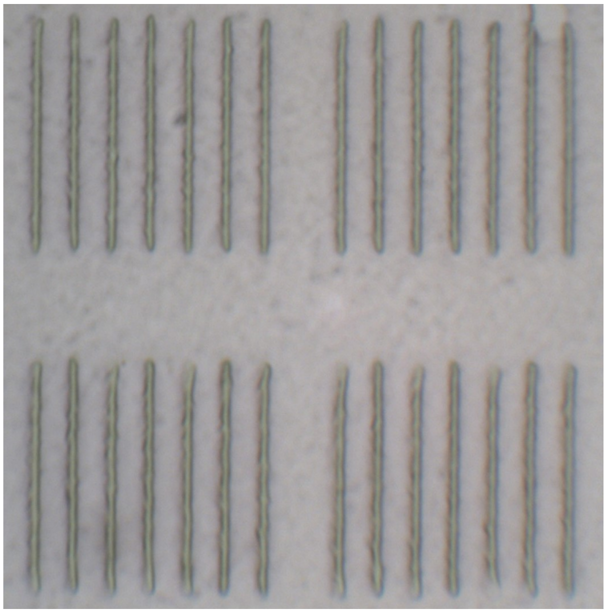

Figure S7. The optical microscope images of Si lines integrating on substrate. The line architectures of Si are tightly attached to substrate. In fact, the bottoms of many line architectures are wrapped in the substrate rather than floating on the surface.

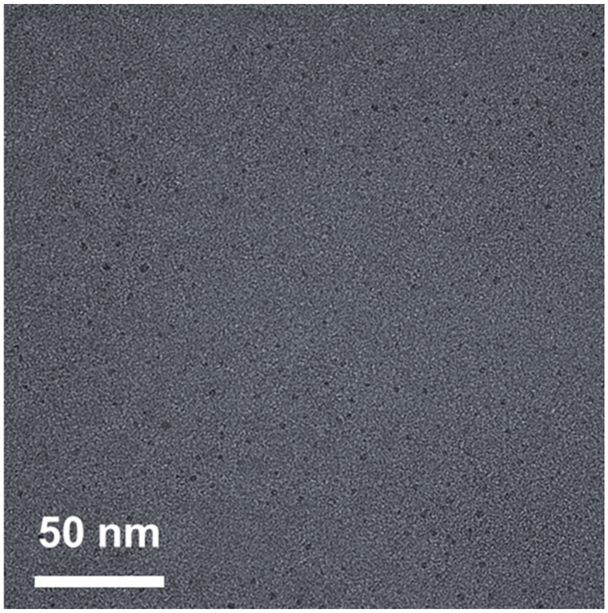

Figure S8. TEM images of Si QDs. These Si QDs origin from the areas between Si architecture.

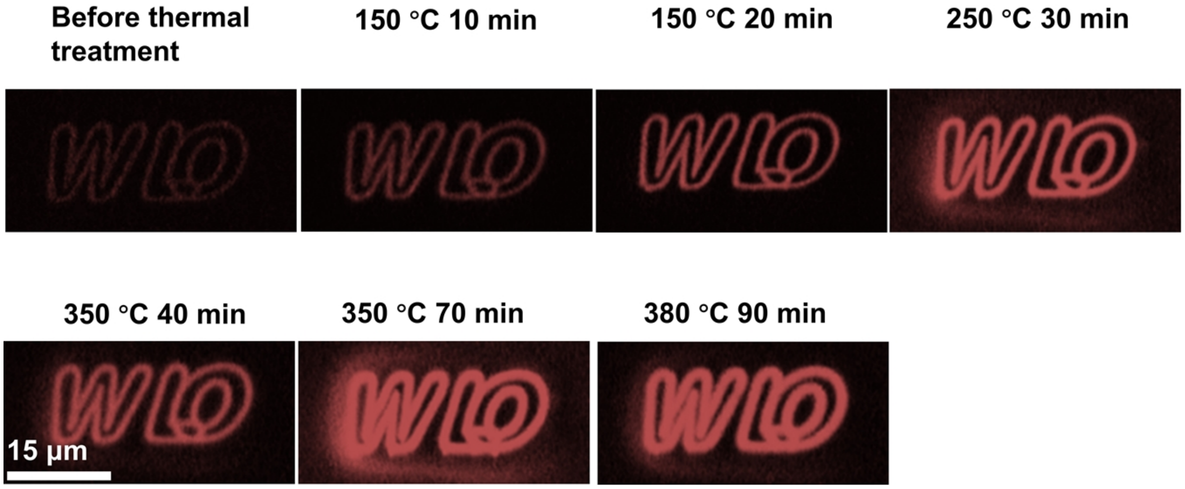

Figure S9. The fluorescence microscopy images of Si architectures before and after thermal treatment at 150, 250, 350, and 380 ºC, the temperature of thermal treatment increases gradually with time (from left to right). After thermal treatment, the Si architectures stacked by Si QDs did not lose the lose their luminescent properties, due to the good temperature stability of Si QDs.

REFERENCES

(1) Peng, Y.; Jradi, S.; Yang, X.; Dupont, M.; Hamie, F.; Dinh, X. Q.; Sun, X. W.; Xu, T.; Bachelot, R. 3D Photoluminescent Nanostructures Containing Quantum Dots Fabricated by Two-photon Polymerization: Influence of Quantum Dots on the Spatial Resolution of Laser Writing. *Advanced Materials Technologies* **2019,** *4*, 1800522.

(2) Fischer, J.; Wegener, M. Three-dimensional Optical Laser Lithography Beyond the Diffraction Limit. *Laser & Photonics Reviews* **2013,** *7*, 22-44.
